# Building and sustaining therapeutic relationships across treatment settings: a qualitative study of how patients navigate the group dynamics of mental healthcare

**DOI:** 10.1186/s12888-025-06874-5

**Published:** 2025-04-28

**Authors:** Henrik Wang Iversen, Henriette Riley, Marit Råbu, Geir Fagerjord Lorem

**Affiliations:** 1 Department of Psychology, UiT The Arctic University of Norway, Tromso, Norway; 2 Division of Mental Health and Substance Abuse, University Hospital of North Norway, Tromso, Norway; 3 Department of Health and Care Sciences, UiT The Arctic University of Norway, Tromso, Norway; 4 Department of Psychology, University of Oslo, Oslo, Norway

**Keywords:** Therapeutic relationships, Mental healthcare, Complex psychosocial needs, Patient-Provider interaction, Patient-Centered care, Stigma, Paternalism, Qualitative methods, Patient empowerment

## Abstract

**Background:**

Therapeutic relationships are vital for patients with complex and long-term psychosocial needs, yet such patients often face fragmented and unstable relationships within mental healthcare. These patients are more often than others moved between treatment settings and caregiving teams. Statutory obligations strain the relationships with frequent hospitalizations adding to the burden This study explores how these patients perceive and navigate therapeutic relationships, highlighting both positive and negative experiences across various treatment settings.

**Methods:**

This is a qualitative study with a narrative approach utilizing in-depth interviews focusing on participants personal experiences and perceptions. We utilized purposive sampling to recruit patients with extensive hospitalization experience, operationalized as more than four admissions within one year or more than four successive weeks of hospitalization. Our sample consisted of 16 participants, twelve women and four men. The interviews were analyzed using a holistic-content approach.

**Results:**

We found that therapeutic relationships were built on healthcare professionals recognizing and addressing patients’ needs and advocating for their interests within the service system. Participants described therapeutic relationships as sources of collaboration, stability, and support but found them challenging to sustain due to fear of rejection and institutional barriers. Successful relationships worked as a vital buffer, offering protection against malpractices and depersonalized care.

**Conclusion:**

Therapeutic relationships play a crucial role in supporting patients with complex needs, but relational dilemmas and malignant group dynamics often impede their development. Mental healthcare services have an ethical responsibility to foster and maintain therapeutic environments and professional cultures that enable personalized care, while maintaining boundaries through reflective practices.

**Supplementary Information:**

The online version contains supplementary material available at 10.1186/s12888-025-06874-5.

## Background

Therapeutic relationships in mental healthcare extend beyond the psychotherapeutic dyad and can vary immensely in terms of goals and duration [1–2]. These relationships can stretch over many years in an outpatient setting or can consist of a brief encounter at an acute psychiatric ward. At best they contribute to continuity of care across diverse treatment settings, but what role do patients with complex and long-term psychosocial needs perceive these relationships to play in their care?

Mental healthcare is interdisciplinary, and a large body of literature describes how different professions define their role and relationship with patients distinctively [3–4]. Common components of the therapeutic relationship across disciplines usually include trust, empathy, respect, collaboration, and support [5–7]. These elements are essential in fostering effective communication and mutual understanding between patients and professionals, contributing to the overall therapeutic process and improving patient outcomes.

Previous research has found therapeutic relationships to play a central role in personal recovery [8–9]. These studies show that collaborative relationships enhance recovery by fostering hope and self-directedness. Personal goals and the assistance of professionals in pursuing these goals are critical to the process [9]. Strong working alliances, where professionals actively engage in recovery-promoting strategies, are associated with better recovery outcomes [8].

For patients facing serious mental illness, substance abuse, unemployment, and social marginalization, relationships with healthcare professionals are often challenging and unstable [10–11]. This subset of patients with complex needs more often than others are moved between different treatment settings and caregiving teams [12–13]. Statutory obligations, such as coercive treatments and community monitoring, strain these relationships further [2], with frequent hospitalizations adding to the burden [1]. Research has found that therapeutic relationships weaken after three or more hospitalizations, indicating this to be a tipping point for when collaboration breaks down [14].

Based on a recent study, Engstrom and colleagues [15] argue that finding ways of offering therapeutic relationships to these patients is an ethical imperative. Still, patients frequently report that their relationships with healthcare professionals are unhelpful or even harmful, citing issues with a variety of professional and services’ characteristics [16–17]. Repeated negative interactions, where professionals hold authority and patients feel vulnerable, can erode patient confidence [18].

The absence of therapeutic relationships is associated with patient disengagement, rehospitalization, increased perception of coercion, and generally negative treatment outcome [14–16]. It is therefore essential to better understand why therapeutic relationships fail. Research have shown that patients with complex needs and extensive hospitalization experience calls for relationships characterized by special attention, exclusivity, and professionals going beyond standard roles and institutional routines [11, 16]. This implies that standard care is not sufficient in meeting their needs.

Mental healthcare has been criticized by both patients and professionals for its paternalistic culture, rigid medical models, and institutional injustices, which strain patient-provider relationships [18–20]. Contributing factors include underfunding, staff shortages, heavy workloads, hierarchical structures, and systemic inefficiencies that hinder personalized care. Despite the centrality of relationships in the creation and amelioration of mental health problems, there is a lack of emphasis on how professional cultures shape service provision in mental healthcare [21–22].

Studies indicate that patients with extensive hospitalization experience and complex needs experience more stigma and prejudice than other patients [23–24]. Koekkoek and colleagues [10, 17] found that healthcare professionals perceive patients as “difficult” when they do not fit within diagnostic categories or standard treatment regimens because of their complex and long-term needs. As a result, patients may anticipate negative labeling and adopt avoidance or non-disclosure strategies, further undermining the therapeutic relationship [18]. This can intensify negative experiences and foster long-lasting distrust toward services.

While the therapeutic relationship has been studied at various treatment settings for different patient groups, there is a knowledge gap concerning how it plays out throughout service provision for patients with complex needs [1, 26–28]. Treatment trajectories for these patients are characterized by relational instability and ruptures, making it especially challenging to study their relationships with healthcare professionals. Undertaking this task may improve treatment outcomes, enhance patient engagement, and promote recovery for a marginalized group of patients [29–30].

Decades of research has shown positive correlation between outcome of treatment and the quality of the therapeutic alliance [31]. This applies in particular for the alliance as it is perceived by the patient [32]. This is a good reason to prioritize patient perspectives in research. Specific conceptual and methodological work in these areas can provide new perspectives on the therapeutic relationship in mental healthcare [7].

In this study we seek to understand how long-term patients with complex needs perceive the role of healthcare professionals in their service provision and what their experiences are with therapeutic relationships. We ask the following research question: What characterize therapeutic relationships for these patients and what strategies do they employ to build and sustain them?

## Methods

### Design

This is a qualitative study with a narrative approach utilizing in-depth interviews focusing on participants’ personal experiences and perceptions [33–34]. The interview guide was semi-structured, with open-ended questions to encourage participants to tell their stories in their own words, offering a comprehensive view of how therapeutic relationships evolved over time. The interviews aimed at identifying both positive and negative encounters.

Our methodology draws on the work of Riesman’s [33] narrative inquiry and Frank’s [34] socio-narrative approach. We focus on how storytelling serves as a form of social communication, enabling individuals to embody different identities and express complex emotions, thoughts, and experiences related to illness and treatment [34]. This is an open approach where the analytical aim was to highlight the patients’ narratives to avoid a theory-driven analysis, emphasizing our participants subjective understanding of therapeutic relationships prior to professional explanations [33].

### Participants

We utilized purposive sampling to recruit patients with extensive hospitalization experience, operationalizing this as having more than four admissions within a year or over four consecutive weeks of hospitalization. We recognize that the definition of “extensive” may vary based on local context. We did not define how recent participants hospitalization experience had to be, thus including participants who were both presently hospitalized and who had not been admitted for many years. This contributed to a rich dataset consisting of people at different stages of treatment and recovery.

We included patients aged above 18 years and excluded individuals that did not speak any Scandinavian language or English, and people that lacked capacity to consent to participation. Our sample consisted of 16 participants, twelve women and four men. We recruited participants whose age ranged from mid 20s to late 50s, who came from diverse backgrounds and whose service experience encompassed different areas of mental healthcare, such as acute psychiatric wards, forensic units, community mental health centers, assertive outreach teams, outpatient clinics, and drug rehabilitation facilities.

Participants self-reported symptoms and diagnosis, and all were found to at one time having qualified as having serious mental illness (SMI), as defined by the US National Institute of Mental Health: “*a mental*,* behavioral*,* or emotional disorder resulting in serious functional impairment*,* which substantially interferes with or limits one or more major life activities*” [35]. However, the concept of severe and persistent mental illness lacks a consensual definition and refers more to a patient population rather than a disease entity, which is in line with our use of the term [36].

### Recruitment

We recruited participants through contact persons within local client networks and the regional mental healthcare division. The first author participated in several staff meetings within the division to inform healthcare professionals about the project and to gain their support in forwarding information to patients who satisfied our inclusion criteria. Contact persons in the various departments then identified patients and approached them with a request to participate.

Potential participants were given informational material with instructions on how to register for the study via an online form. After registration, participants were contacted by the first author to arrange the time and place for the interview. This ensured that the contact persons did not know who had registered or declined, and the researchers did not know who had been asked but chose not to participate.

Local mental health client networks distributed information about the project using their social media accounts and mailing lists. Information material was spread at locations that individuals with extensive hospitalization experience were known to frequent such as club houses. These participants registered through the same online form as the above. We completed our recruitment process after a one-year period.

### Interviews

The interviews, conducted by the first author, aimed to capture participants’ positive and negative experiences with treatment and care. They focused primarily on participants’ personal narratives, exploring their experiences within the healthcare system. The interviewer encouraged open storytelling while minimizing directional steering in the conversation [37].

Interviews were conducted at various locations in the hospital, the university, offices of the assertive outreach team, and in one instance at a participant’s home. The duration of the interviews varied, with most lasting approximately an hour and a half, some extending two hours, and a few concluding in less than one hour. All interviews except for one were conducted one on one with the participant. The exemption was a participant who asked to have her contact person at a hospital ward present during the interview for emotional support.

The interview guide was specifically designed for this study. It started with open-ended questions such as “What are your experiences with mental healthcare?”. This naturally prompted participants to share stories about specific times, people and places. Their relationships with healthcare professionals where a particular emphasis in these stories, facilitated by more specific questions by the interviewer such as “How did that relationship affect you?”.

### Analysis

The interviews provided us with rich and nuanced data in the form of comprehensive stories about participants’ service experience. We recorded and transcribed the interviews verbatim and analyzed them using a holistic-content approach. Throughout the analysis we focused on the different meanings of what the participants were communicating, trying to recapitulate their way of seeing the world, asking whether there were connections between events, the participants and the wider context. This open approach is particularly suitable for developing theoretical arguments from participants stories and invites the reader to think beyond the obvious in the text, creating space for interpretation [33].

The first step in analyzing the interviews involved reading the transcripts and discussing emerging questions about the material within the project group comprising all co-authors. Next, we entered the data into NVIVO, a software application designed for effective management of qualitative data [38]. We employed first-cycle coding to identify and summarize data relevant to our research questions. Initially, the codes were descriptive, derived directly from the content of the participants’ talk during the interviews.

Next, we applied second-cycle pattern coding to organize our material into themes and concepts across participants [39]. During this process, the researchers reflected on general questions such as: “What is going on in the participants’ stories?” and “What role do relationships play in their service provision”. We created a coding tree and wrote analytical memos for all potential themes identified during the analysis [39]. This process involved delving into each interview to capture the essence of the participants’ stories and identifying overarching narratives across interviews.

Our post-coding analysis further refined our themes and established the final structure of our findings by contextualizing the data with relevant research literature and engaging in further discussions within the project group. Therapeutic relationships materialized as a main theme in the participants’ stories. We actively looked for stories that nuanced and contrasted each other to ensure sufficient range in our findings. All findings were cross-validated and discussed in the project group until agreement.

### Ethical considerations

The Norwegian Agency for Shared Services in Education and Research (NSD) approved the project in April 2022 (approval no: 783719). The research ethics committee of the Department of Psychology, UiT The Arctic University of Norway approved the project in May 2022. The project was conducted in accordance with the 1964 Helsinki Declaration and its later amendments. We took care to discuss confidentiality in detail before the interview started. Data was anonymized immediately after transcription and stored securely on password-protected servers accessible only to the research team. All identifiable data will be destroyed after a period of five years, in accordance with institutional policies.

Written informed consent was obtained from all participants, and confidentiality was discussed in detail prior to the interviews. The interviewer was attentive to the participants’ capacity to consent to participation throughout the interview. If the participants’ capacity came into question, the interview was concluded gently, followed by an assessment as to whether to use the data provided. We strove to reduce the power imbalance between participant and interviewer through open discussions about the aim of the study, the nature of the questions, and by soliciting feedback about our interpretation of their responses [26].

## Results

We found that relationships were built by healthcare professionals recognizing and addressing participants’ needs, supporting and advocating for them within the service system over time. Such patient-centered relationships provided a buffer, offering protection amidst otherwise negative experiences in mental healthcare. Participants made considerable efforts to hold on to these relationships but found them challenging to sustain due to fear of rejection and institutional barriers.

Failed relationships were marked by disappointments, transgressions, neglect, and paternalistic treatment, ultimately reinforcing feelings of distrust. Participants often described their experiences in polarized terms, either idealizing professionals or antagonizing those they perceived as failing them. These failures were intensified by malpractices which enabled depersonalizing treatment. However, when having a strong relationship in place, participants felt empowered, highlighting the critical role of therapeutic relationships in mitigating patients discontent with the broader mental healthcare system.

### Having ones’ needs recognized and supported

Participants described how therapeutic relationships were built and sustained over time by healthcare professionals recognizing, addressing and supporting their needs within the healthcare system. They emphasized the importance of healthcare professionals acting as their advocates by taking their side and giving them a voice. This was especially vital in light of their many negative experiences with services in general. One participant described her encounter with a new member of her treatment team at an acute psychiatric ward, where she had been admitted repeatedly over the years:

*“She is the first therapist I’ve had whom I feel was skilled. I felt that she saw me and genuinely wanted to collaborate with me. Even though I was in a bad state*,* we had good conversations. She also saw that*,* despite being very unwell*,* I was trying to accept help in my own way*,* to varying degrees. She wasn’t someone who just went along with whatever I said. She looked me straight in the eyes*,* and if I said something completely off-base*,* she called me out on it. That’s how I want to be treated*,* even without asking for it*,* and it felt very genuine to me. It meant a lot that*,* in the last month of my hospitalization*,* they let me be there voluntarily and show that I could accept help willingly. For me*,* that was very important.” (Agnes)*.

This participant describes how her therapist understands her by recognizing her efforts to accept help while also challenging her illness-related behavior. This results in her “being seen” as a person beyond her illness, which she perceives as genuine support of her agency, rather than being relegated to a passive patient role. This experience of being understood by a therapist resonate with other participants’ narratives. However, the confrontational approach favored by this participant may risk offending others. Employing a direct challenging style requires a deep understanding of the patient’s personal preferences which is typically acquired over time.

The participant also noted that her therapist allowed her to stay at the ward voluntarily, which likely involved resisting pressure from colleagues for early discharge. In this way, therapeutic relationships were described as a positive departure from the typical barriers and restrictions encountered in mental healthcare. The emphasis on therapeutic relationships representing a positive break from negative experiences with services in general were elaborated on by other participants:

*“I would go so far as to say that psychiatry ruined my life. But then I started therapy with the psychologist I still have*,* and I’m not letting her go. So now we only have maintenance sessions 1–2 times a year just so I don’t lose her. I don’t need to see her that often*,* but she must be there. She is my*,* what’s it called? My anti-anxiety drug. (…) She has allowed herself to be critical of the system regarding how I’ve been treated. I felt like I got support there.” (Tone)*.

Participants in therapeutic relationships expressed deep affection for healthcare providers who offered them protection and security from what they perceived as malpractices of mental healthcare, which for this participant had “ruined her life.” After years in and out of institutions and under heavy medication, establishing a therapeutic relationship with a psychologist was key to breaking this cycle. Whether such regimens should be considered malpractice remains contested, given that hospitalization and medication are sometimes indispensable for certain patients. However, this participant strongly argued that her case amounted to malpractice. This polarization between positive and negative experiences was common among participants and may reflect inherent tensions within mental healthcare.

In any event, the psychologists’ willingness to criticize the system and take the participants’ side was crucial in building a trusting relationship. Therapeutic relationships empowered participants to take a more active role in treatment by ensuring their needs and preferences were recognized, supported and advocated for by a healthcare professional. Ultimately, this illustrates how quality of services can hinge on which professional a patient encounters and whether the professional is prepared to go beyond their traditional role by, for example, criticizing the system.

### Hanging on to therapeutic relationships

Therapeutic relationships were described as rare opportunities for participants that they had to hang on to within a system that did typically not facilitate for such relationships. Having encountered a professional with whom they believed a therapeutic relationship could be established, participants endeavored to prolong their contact, viewing these relationships as vital lifelines:

*“Throughout my entire medical history*,* I’ve followed this principle. If you find someone who is skilled and listens to what you have to say*,* hold on to them. Just like I held on to [name of psychiatrist] (…) Especially after he apologized for discharging me the first time. I’m not interested in suing him because he made a mistake. I mean*,* everyone can make mistakes. But he has taken responsibility and since then*,* I’ve had him as sort of a safety person. I have his mobile number. If I’m in contact with the crisis resolution team or if the emergency room doesn’t want to admit me*,* they can call him*,* and he can say*,* ‘Listen here.’ As chief psychiatrist he can override them.” (Irene)*.

Relationships were placed at the center of service provision by many of our participants. While “being seen” was important to some, for this participant, it was crucial to “be heard”. Participants were willing to look past previous grievances if their provider appeared genuinely apologetic, as the psychiatrist in this example. Giving out his private number was effective in strengthening the participants’ trust in him. It is also possible that previous ‘mistakes’ made the psychiatrist more inclined to give out his number to this particular participant. Standing up to colleagues or going against hospital routines further fortified these relationships and made the participants feel protected. This signaled that providers were not bound by loyalty to the institution but could be loyal to the participants. This psychiatrist’s authority allowed this participant to challenge decisions, effectively lending her his “voice” and empowering her. Like others, she became deeply invested in this relationship and became desperate when she felt their mutual understanding was dissolving:

*But yes*,* I actually thought about an overdose again later*,* when I felt that [psychiatrist’s name] didn’t understand me. I had brought all my medications to an appointment with him. I’m not sure if I explained to him how I was feeling*,* but somehow*,* I felt understood*,* like we could work things out. Because of that*,* I was able to take my medications back home and avoid doing it again. (Irene).*

This illustrates how even longstanding therapeutic relationships can falter. External circumstances may play a role, yet in this case the participant’s sense of a dissolving understanding possibly reflects her internal state as much as an actual rupture. It is also possible that the relationship was not built on such a solid foundation as initially presented; her remark about not wanting to sue him for past mistakes can be interpreted as a subtle threat. Suicidal ideation may also be understood as a desperate way for patients to gain access to treatment and care.

Patients with extensive hospitalization histories often have experience with relationships that end abruptly or turn bad, which in turn may affect how they relate to healthcare professionals. In this case, the participant’s impulse to take an overdose when she felt misunderstood suggests that the action served a communicative function, akin to raising one’s voice. As such it represents a powerful negotiation tool and a way to put pressure on the professional in addition to being an expression of the desperation patients feel when therapeutic relationships, they depended on are breaking down.

### Frustration, disappointment and distrust

Participant narratives detailed frustrating and disappointing experiences with failed relationships. These failures were characterized by misunderstandings, conflicting perspectives, offences and transgressions. Healthcare professionals were often unsuccessful in addressing participants’ needs, perceived as overstepping boundaries, misattributing intentions to the participants and siding with the mental healthcare system against them. A common theme was that healthcare professionals should have done more for the participants in a given situation:

*“I didn’t like the psychiatric nurse I was assigned*,* so I reported it after 15 consultations. I tried to get him to do things for me that I wanted*,* but he couldn’t do them (…) One of those things was to have my emergency plan included in my medical record. I eventually got that. Another was to get a patient-controlled admission contract. I didn’t get that. And the third was to stop taking medication. He couldn’t do that either. So*,* it was agreed that I would have a medication review by someone else. It was a psychiatrist*,* but she was terrible. She made me cry during the consultation. So*,* I asked to get a new therapist.” (Mia)*.

The relationship with a psychiatric nurse was frustrating because he was unable to deliver the services this participant requested, resonating with other participants who recounted similar unsatisfying experiences. A fear of personal rejection often surfaced when asking for help, highlighting the high stakes involved for our participants. This situation also reveals how differing expectations can contribute to ambiguity and confusion in the relationship.

For this participant, the final straw was when the nurse failed to protect her from the consulting psychiatrist, which undermined her trust and ultimately led her to terminate the relationship. The nurse is portrayed as weak and ineffective, in stark contrast to the authority exhibited by some healthcare professionals in previous examples. This discrepancy underscores the participants’ acute awareness of professional hierarchies and their tendency to adopt stereotypes about their providers. Other participants described their healthcare professionals in an even more antagonistic manner:

*“I don’t know if he was inspired by Freud or something*,* but he had this huge chair that he sat in himself*,* while the staff had to sit on these small chairs*,* and I had to sit on the edge of a bed. He attributed opinions and characteristics to me that I had never expressed. I didn’t understand where he got those things from. I had never said anything remotely close to that*,* nor anything that could be interpreted in that way. He was an arrogant jerk. (Lisa).*

Our participants recounted numerous instances of failed therapeutic relationships, expressing both indignation and critique regarding their treatment. These failures were often attributed to professionals who did not listen, care, or accurately interpret their needs, thereby hindering relationship-building and fostering distrust. This particular participant did not recognize herself in the psychologist’s interpretations and felt offended by him. Her sarcastic reference to Freud and the seating arrangement underscores her stereotypical view of the psychologist in the absence of a genuine relationship.

It is quite possible that the psychologist behaved insensitive or even reprehensible; alternatively, he may have attempted to challenge her illness-related behavior. Whether such interpretations are effective depends on numerous factors. However, such interventions should be undertaken with caution in non-traditional psychotherapy settings. The presence of staff as an audience likely heightened the participants apprehension, contributing to the asymmetry and inferiority she experienced in the situation. These sentiments are echoed by another participant’s antagonistic relationship with a psychologist working at a crisis resolution team:

*“But the psychologist I went to was not kind. She might be skilled in her field*,* but she was so afraid that we were going to be institutionalized that we shouldn’t be admitted at any cost (…) She had diagnosed me with mixed and other personality disorders and said that it neither could be treated or medicated. She thought I was faking it because I wanted disability benefits” (Irene).*

This participant, who had frequent contact with a crisis resolution team, described the psychologist working there as “not kind.” This perception was partly due to the psychologist questioning her intentions and failing to offer any hope for future recovery. However, her devaluation may also reflect her repeated experiences of rejection by the services. Crisis resolution teams aim to prevent unnecessary hospitalizations, which can conflict with the perceived needs of patients in crisis.

Participants described how healthcare professionals siding with the services against their interest left them feeling disregarded or even harassed. It was a reoccurring theme in participants narratives that they were misunderstood or had opinions and characteristics attributed to them which they did not identify with, and which had negative consequences for their access to services. This resulted in antagonistic relationships characterized by mutual distrust and misattribution.

### Encountering barriers to developing relationships

Participants pointed to several barriers, typical for the institutional setting, that hindered therapeutic relationships from developing and them from being known in a personal way by their providers. This included hierarchical structures and nonintuitive division of tasks, stigma and lack of resources. Participants also described how they felt judged based on their treatment history, further cementing their patient identity. This resulted in relationships that were impersonal, simulated or pretend:

*“I don’t have the impression that there is a lot of collaboration. For example*,* if I was talking to a nurse*,* they could agree that I was having a hard time and acknowledge my feelings and all that*,* but as soon as it came to the question of ‘Should it be this way?‘*,* it was always ‘You need to discuss that with the doctor*,* because that’s something you and he have to deal with*,* and he handles all the medical aspects and diagnoses.” (Tone)*.

In this example, the nurse, though seemingly empathetic, disclaimed responsibility when the participant questioned her treatment. This made the relationship feel instrumental, not genuinely supportive. Participants also talked about the relationships between different healthcare professionals in their caregiving teams, often emphasizing the hierarchical power distribution between them. The participant in this example possibly knew that it was the doctor she ultimately had to talk to, as he had last say over treatment decisions, but may have hoped to find an ally in the nurse to support her before approaching the doctor with her concerns. The nurse appears unwilling to provide this kind of support, thus rejecting her attempt at establishing a therapeutic relationship.

Although professional hierarchies in mental healthcare exists for a reason, they appear overly rigid from our participants’ perspective, hindering the negotiation of genuine therapeutic alliances. One participant, who had been repeatedly admitted to the same acute psychiatric ward over many years, shared her experience:

*“You are judged based on their previous experiences with you. That’s what I feel a bit like when you come to a ward like me*,* who has been in and out since I was 16. You know many of the staff*,* and they only see you when you’re at your worst*,* when you’re almost not yourself. So*,* it’s as if they don’t understand that the person you are in there is not the person you are outside for the rest of the year. You function in society*,* you have a life*,* you have a job*,* and you do those things. You come there when you’re in an extreme crisis*,* you know?” (Agnes).*

This participant believes that healthcare providers’ knowledge of her from repeated admissions limit their ability to ‘see’ and understand her beyond her patient identity. Contrary to previous examples, where knowledge fostered a more personal connection, in her experience, it can serve to confine her to a restricted patient role. In the institutional setting she describes, there is no one for her to negotiate a therapeutic relationship with, only nameless “staff” in whose reflection she is reduced to only a patient.

Establishing therapeutic relationships during acute phases is inherently challenging. Notably, this participant described in an earlier vignette how her prolonged stay at the acute psychiatric ward eventually enabled her to develop a therapeutic relationship with her new therapist. The opportunity for continuity in care beyond the acute phase can be pivotal in fostering relationships that allow patients to be recognized as whole individuals.

### Transgression, neglect and loss of humanity

The participants conveyed that treatment that did not originate from therapeutic relationships felt impersonal and patronizing. In the absence of therapeutic relationships, treatment could lose its humanity and become transgressive or neglectful. This absence could be the result of healthcare professionals rotating frequently, leaving little opportunity for consistent contact:

*“I had four or five doctors during that period. And then a doctor I had never met before would just come in and say*,* now we’re doing it this way. (…) They had a bit of a mafia-like style. ‘If you don’t go there voluntarily*,* you’ll go there involuntarily.’ ‘If you don’t want to take the medication*,* then you’ll get it through injection.’ You gave in very quickly.” (Sara)*.

For this participant, hospitalization was marked by unstable relationships with doctors. Other participants reported similar issues, citing frequent staff turnover and limited time and resources as barriers to cooperation and relationship-building. When treatment did not originate from a relationship, patients’ needs and wants were not known by a significant other. This contributed to giving their service provision a depersonalized and paternalistic quality. This participant characterizes the pressure from healthcare professionals, somewhat stereotypical, as “mafia-like” intimidation. While such intimidation techniques may be common in mental healthcare, they were perceived as offensive by our participants, especially when there was no therapeutic relationship in place.

This illustrates how providers who probably have the best of intentions end up playing the role of antagonists towards patients they have not had the time or opportunity to get to know in a personal way. It also mirrors the pressure and persuasion patients themselves desperately engage with when struggling to access treatment and care. This situation poses a threat to the humanity of both patient and provider:

*“They [the staff] have lost their humanity. Every patient is someone’s brother*,* sister*,* mom*,* or dad. They are working with people; we are not just objects or animals. But sometimes you can get that impression when you are put in seclusion and spend three weeks in a room with different people coming in two or three times a day. And if that’s all they have to offer*,* it’s not really treatment.” (Agnes)*.

Participants’ sense of self can diminish when subjected to invasive treatment such as coercion or seclusion, particularly when these interventions are carried out without emotional support or recognition of their distress. Service provision characterized by management and control, rigid structures, risk management, and procedural compliance can limit the time and emotional capacity healthcare professionals have for relational engagement. Our participants described that in the absence of therapeutic relationships, both their own sense of humanity and their sense of healthcare professionals’ humanity were lost. This resulted in stigma and stereotypical thinking.

Therapeutic relationships were perceived by participants as a potential safeguard against the depersonalizing aspects of institutional care. Alas, repeated hospitalizations, staff turnover, systemic barriers, rejections and neglect fostered distrust among participants, preventing the development of such relationships. This left them unprotected from injustices therapeutic relationships might have helped prevent.

## Discussion

This study found that patients with complex needs and extensive hospitalization experience regard therapeutic relationships as vital to their care. These relationships serve as a protective buffer against the adverse effects of mental healthcare. However, systemic barriers and the inherent fragility of these relationships make them challenging to establish and sustain, often leading to depersonalized treatment. In the discussion that follows, we examine how these challenges intersect within the intricate relational and at times antagonistic dynamics of mental healthcare systems. Ultimately, we argue that services bear an ethical responsibility to foster therapeutic environments that cultivate personalized, relationship-centered treatment and care.

### Challenges in building and sustaining therapeutic relationships

Patients with extensive hospitalization histories appreciate when healthcare professionals’ step beyond formal roles and routines to build trust, seeing this as a sign of genuine, personalized care [11]. However, in resource-constrained environments, professionals struggling to meet basic expectations are unable to exceed their roles, putting patients who rely on such efforts at a disadvantage [16]. Under these conditions, patients often feel neglected, a sentiment voiced by our participants [1].

Healthcare professionals’ authority was appreciated when it empowered and benefited participants. Lending the ‘voice’ of their healthcare professionals ensured that our participants were heard when they expressed their needs. Research indicates that patients are more willing to accept professional authority and acknowledge their own dependency when treatment is grounded in a therapeutic relationship [40–41].

When there is no relationship at the foundation of treatment, pressure and subtle coercion is more likely to be experienced as ‘mafia-like intimidation’. However, the fragility of therapeutic relationships makes it challenging to balance professional integrity, genuine support, and assertion of authority, all at the same time, often leading to relationship failures [1, 5]. Our study illustrates the dilemmas healthcare professionals face in balancing these considerations. Sharing private contact details, while fostering trust, risks privatizing the relationship.

The balance becomes even more difficult when patients have unmet dependency needs, as they are particularly vulnerable to both neglect and overdependency [10, 17]. Deep-seated fears of abandonment and rejection can lead patients to interpret professional boundaries as personal slights, escalating into feelings of harassment and persecution [10]. Attempts at persuasion and defensive acting out, such as threatening suicide, can strain the therapeutic relationship, placing considerable emotional demands on professionals, further complicating the care process [21].

Participants expressed a desire for relationships with professionals who had the authority to make decisions that impacted their treatment. However, day-to-day interactions at wards are often delegated to lower-level staff, as seen when a nurse disclaimed responsibility for a participant’s concerns. Because services are not always structured to support therapeutic relationships, healthcare professionals wishing to engage in such relationships may need to challenge hospital routines and hierarchies, creating additional dilemmas [16].

Therapeutic relationships were thus shaped by how far healthcare professionals were willing to go beyond formal roles and hospital routines. However, the inherent fragility of these relationships made them susceptible to failure and going beyond formal roles and routines can be problematic for several reasons. This underscores the need to shift focus from the individual dyadic relationships to the broader therapeutic environment in which they are situated and constituted.

### Navigating the group dynamics of mental healthcare

Limited resources, heavy workloads, rigid hierarchies, and strict procedures can alienate patients and professionals alike, and undermine therapeutic relationships [1]. Many treatment teams suffer from unclear boundaries, conflicting objectives, and professionals on short-term rotations, leading to tensions arising from differing perspectives, shifting responsibilities, and contested hierarchies [21].

Problems manifest when professionals’ emotional capacity is overwhelmed by service demands, expectations and patients’ projection of their inner turmoil onto the treatment setting. When professionals attempt to navigate these challenges by going beyond their formal roles, routines and hierarchies, they may unintentionally contribute to splitting dynamics by offering support based on strong identification with the patient [10].

Patients with extensive hospitalization histories who frequently experience inconsistent care may idealize professionals who advocate for them while antagonizing those who uphold service constraints. This dynamic can create internal conflict and divisions within treatment teams, where colleagues, consciously or unconsciously, mirror these polarized perceptions, reinforcing an us-versus-them mentality [42].

Splitting contributes to defensive coping mechanisms becoming entrenched within team dynamics [17, 21]. According to Bion [42], group dysfunction arises when unresolved tensions within a team result in professionals colluding in rescue fantasies, unconsciously adopt a passive stance, expecting an authoritative figure to provide all solutions or indulge in scapegoating and other antitherapeutic processes.

The labeling of patients as difficult, is a form of scapegoating indicated by our participants, that hinder the development of therapeutic relationships and can lead to unnecessary coercion or premature termination of treatment [17]. Our participants sought allies among healthcare professionals to protect them from such negative reactions, but paradoxically this may serve to only strengthen splitting and scapegoating dynamics.

While intervening against scapegoating can be ethically justified, such actions also risk escalating conflict. In today’s system, healthcare professionals may struggle to fully embody the role of genuine helpers unless they are willing to advocate for their patients, even when this means challenging their employer or colleagues. When they avoid this challenge, the “difficult” patient, who feels unseen, unheard, and unrecognized, is mirrored by healthcare professionals who remain unacknowledged as true helpers [10].

Therapeutic relationships for patients with extensive hospitalization experience is situated in mental healthcare systems which are prone to depersonalized treatment. The solution to this is not for professionals to go beyond formal roles, privatizing relationships or bearing the sole responsibility for standing up against problematic institutional practices. Service systems must be held accountable for creating therapeutic environments and professional cultures that support personalized treatment and care, are open for negotiating therapeutic relationships, while maintaining professional boundaries [21–22].

Campling [21] argues that mental healthcare systems fail to adequately address the emotional demands of healthcare professionals’ interactions with patients. Therapeutic environments and treatment teams are vulnerable and can quickly unravel and become malignant if they are not properly understood and managed [1, 21]. This requires supervision and fostering reflective practices where healthcare professionals can confront the dilemmas, barriers and group processes affecting therapeutic relationships. Our study highlights how this ethical responsibility is often neglected in fragmented service systems.

### Strengths and limitations

By exploring the experiences of patients with complex needs across treatment settings this study builds on and adds to existing knowledge about therapeutic relationships in mental healthcare [1–2, 25]. Our research design and use of narrative methods proved especially useful in gathering composite and authentic stories, and we are confident that our findings represent an important contribution to the research field. We furthermore argue that recruiting patients who represent a minority at the extreme end of the mental health continuum allows us to highlight problems that are universal in nature, although they may exist only to a weaker extent in better-off patients. Still, a limitation of the study is that our only source of information is interview data, and we can only make inferences about participants’ subjective experiences of the relationships, not how providers perceive them.

Considering that our participants are often difficult to recruit for interviews it is a strength with our study that we succeeded in recruiting and interviewing 16 patients. By not defining how recent participants hospitalization experience had to be we were able to include more participants than if we only recruited participants in active treatment. This however increased the retrospective nature of our participants’ narratives and assessments which could be considered a limitation as certain experiences are highlighted, and others are forgotten in hindsight. This thus introduced a potential recollection bias which the authors had to be conscious about throughout the research process [39].

## Conclusion

Therapeutic relationships for long-term patients with complex needs are vital to service provision yet challenging to build and sustain across treatment settings. These relationships offer essential support and protection against negative effects associated with the mental healthcare services such as paternalism and scapegoating. However, they often falter due to fear of rejection, institutional barriers, relational dilemmas and malignant group dynamics. Services have an ethical obligation to foster therapeutic environments and professional cultures that enable personalized care, while maintaining boundaries through reflective practices that address the challenges and barriers to building effective therapeutic relationships.

## Electronic supplementary material

Below is the link to the electronic supplementary material.

Supplementary Material 1

## Data Availability

No datasets were generated or analysed during the current study.

## Abbreviations

NSD: The Norwegian Agency for Shared Services in Education and Research
SMI: serious mental illness
