# Supplementary material for: Building and sustaining therapeutic relationships across treatment settings: a qualitative study of how patients navigate the group dynamics of mental healthcare
Source: BMC Psychiatry. 2025 Apr 28;25:424. doi: 10.1186/s12888-025-06874-5 (PMC12036144; doi:10.1186/s12888-025-06874-5)

**Interview**

**Introduction:** Provide a brief introduction to the project and the interviewer. Invite the participant to talk about themselves. Explore the participant’s thoughts on what constitutes a good life and what a normal life means to them. Suggested questions:

- What is well-being?
- What is psychological pain, and when does it arise?
- What does it mean for you to become healthy?

**Well-being Diagram:** Introduce the well-being diagram and suggest that the participant starts drawing their graph from a point where life was stable. Once the participant has drawn their graph, ask them to explain why it looks the way it does. Ask specific questions about turning points on the graph, whether ups or downs. Suggested questions:

- What happened here?
- How did you handle it?
- What could have been done differently?
- What does it take for a person to reverse a negative process and improve their well-being?
- At what points on the graph did you want assistance from the healthcare system, and when did you not want it?
- What kind of help did you receive?

**Treatment Experience:** Explore the participant’s relationship with treatment in general, including both hospitalizations and long-term outpatient or mobile follow-up care. Inquire specifically about institutional experiences. Suggested questions:

- What does good treatment mean to you?
- How does treatment take place?
- What are your expectations of treatment and services?
- What are your experiences with mental healthcare?
- What experiences do you have with seeking treatment on your own?
- Have you ever wanted help but didn’t receive it?
- What experiences have you had with being pressured or forced into treatment?
- What is your relationship with [Hospital's Name] (as an institution)?
- How were you treated during hospitalization?
- Did you meet anyone who had a positive or a negative influence on your treatment?
- What does the word "institution" make you think of?


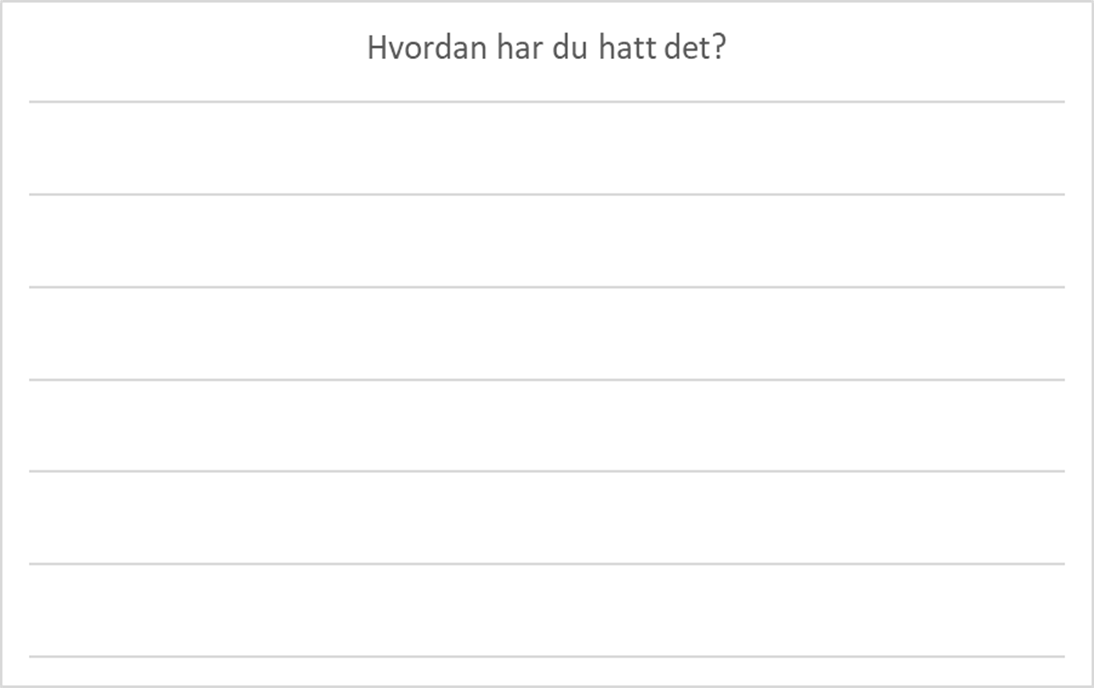

Supplement: Supplementary file 1 — Supplementary Material 1 [file 12888_2025_6874_MOESM1_ESM.docx]
